# A Simulation Method for Underwater SPAD Depth Imaging Datasets

**DOI:** 10.3390/s24123886

**Published:** 2024-06-15

**Authors:** Taoran Lu, Su Qiu, Hui Wang, Shihao Zhu, Weiqi Jin

**Affiliations:** MOE Key Laboratory of Optoelectronic Imaging Technology and System, Beijing Institute of Technology, Beijing 100081, China; 3120210536@bit.edu.cn (T.L.); 3120210606@bit.edu.cn (H.W.); 3220230680@bit.edu.cn (S.Z.); jinwq@bit.edu.cn (W.J.)

**Keywords:** underwater imaging, single-photon imaging, SPAD camera, simulation, dataset

## Abstract

In recent years, underwater imaging and vision technologies have received widespread attention, and the removal of the backward-scattering interference caused by impurities in the water has become a long-term research focus for scholars. With the advent of new single-photon imaging devices, single-photon avalanche diode (SPAD) devices, with high sensitivity and a high depth resolution, have become cutting-edge research tools in the field of underwater imaging. However, the high production costs and small array areas of SPAD devices make it very difficult to conduct underwater SPAD imaging experiments. To address this issue, we propose a fast and effective underwater SPAD data simulation method and develop a denoising network for the removal of backward-scattering interference in underwater SPAD images based on deep learning and simulated data. The experimental results show that the distribution difference between the simulated and real underwater SPAD data is very small. Moreover, the algorithm based on deep learning and simulated data for the removal of backward-scattering interference in underwater SPAD images demonstrates effectiveness in terms of both metrics and human observation. The model yields improvements in metrics such as the PSNR, SSIM, and entropy of 5.59 dB, 9.03%, and 0.84, respectively, demonstrating its superior performance.

## 1. Introduction

Ocean exploration technology has always been an important strategic goal for technology enterprises around the world, enabling humanity to better understand the oceans and discover, develop, and utilize marine resources. In recent years, underwater machine vision has seen rapid development in the field of ocean exploration. The information carried by underwater images can be used to objectively and efficiently display real deep-sea scenes, greatly facilitating the exploration of the underwater world. Images obtained through underwater optical imaging have a high resolution and carry a large amount of information, making them widely applicable in areas such as ocean energy exploration, underwater rescue, marine environmental monitoring, and maritime military operations [1].

In some highly scattering environments, such as water and fog, achieving high-resolution optical imaging remains a major challenge within the photonics community. Particularly for 3D imaging and motion target tracking, the highly scattering environment severely affects the quality of the images. The main limitations in imaging through scattering media are caused by absorption and scattering, leading to the significant attenuation of signals over short propagation distances. Currently, there are many techniques available to distinguish the scattered light returning from the target. In particular, SPAD TCSPC has led to the development of high-performance laser radar and depth analysis devices for highly scattering environments [2].

With the rapid development of single-photon detection devices, single-photon avalanche diodes (SPADs) have gradually become one of the key representatives. They are applied in the field of depth imaging using time-of-flight techniques, achieving remarkable imaging results [3]. Therefore, many scholars have utilized SPAD cameras for research on through-scattering medium interference and underwater imaging. In 2019, Tobin et al. [4] utilized a time-correlated single-photon detection system based on SPADs to investigate the depth imaging of objects through various obscuring media with different densities (water mist, ethanol vapor, and combustion smoke). They obtained depth and intensity data for target imaging up to a distance of 24 meters. In the same year, Maccarone et al. [5] proposed three-dimensional imaging through underwater scattering media using a SPAD array. In their experiment, they utilized a 192 × 128 SPAD detector array, with each pixel integrating Time-Correlated Single-Photon Counting (TCSPC), with an active area of 22 μm^2^ and a fill factor of 13%. The timing resolution of the sensor ranged from 33 ps to 120 ps, and the distance resolution was in the millimeter range. This work represented the first application of SPAD detector arrays in underwater imaging, enabling single-photon depth and intensity analysis at binary frame acquisition rates exceeding 500 Hz under highly scattering conditions. According to the final results, Maccarone et al. achieved three-dimensional imaging underwater up to a distance of 1.7 meters in highly scattering environments. Four years later, in 2023, Maccarone et al. [6] introduced a new underwater SPAD system which is fully submerged with a waterproof sealed compartment. Based on this system, they achieved real-time imaging at 7.5 AL in turbid water. In 2021, Huang et al. [7] used a SPAD detector to achieve real-time underwater optical communication. They employed a simulation method to obtain underwater optical communication data. While simulation is a popular approach for studying SPAD detectors, many simulations focus on the response characteristics of SPADs, which are computationally intensive and make it challenging to generate large SPAD depth image datasets [8,9,10].

Although many researchers have made significant progress in the field of underwater SPAD imaging, there are still some difficulties hindering further research. Firstly, the high cost and time-consuming nature of SPAD fabrication make it difficult to achieve. Secondly, when SPAD cameras are used for underwater imaging, complex sealed housing designs are required, which pose a high risk. Most importantly, conducting underwater imaging experiments is highly challenging, making it almost impossible to acquire a large quantity of diverse, labeled underwater SPAD data through real-world captures. Therefore, we propose a simulation approach that combines virtual environment software, enabling the batch acquisition of diverse, labeled data that closely resemble real SPAD data.

## 2. Theory of Underwater SPAD Imaging Simulation

### 2.1. Underwater Imaging Model

**Figure 1:**
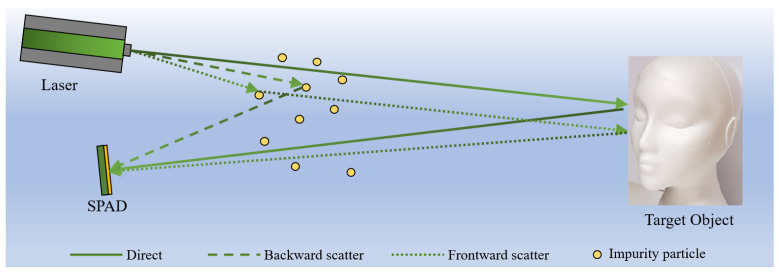
Diagram illustrating the physical model of underwater imaging.

The harsh imaging conditions underwater include the strong absorption characteristics of water towards light signal energy, as well as the scattering effects of the impurity particles in water on light (as shown in Figure 1), posing significant challenges to underwater optical imaging. They introduce a large amount of image noise and blur, leading to decreased image contrast, the loss of target information, and other issues, severely impacting the subsequent identification and analysis of targets [11].

In 1990, Jaffe et al. [12] proposed a model for the attenuation of underwater irradiance:

(1) $$E\left(d\right)=E_{0}e^{\left(-\beta d\right)}$$

where *E* represents the light power at a certain position in water, $E_{0}$ is the light power emitted by the light source, β is the attenuation coefficient of water, and *d* is the distance from this position to the light source. In the process of traveling from the light source to the surface of the target object, some photons do not undergo reflection from particles in the water but directly reach the surface of the target object and, after reflection from the target, return to the camera. Some photons undergo small-angle reflection from particles in the water, and some photons undergo large-angle reflection from particles in the water and return directly to the camera after reflection. We refer to the portion of light that has not undergone reflection from water particles during photon transmission as the direct component. The light that undergoes small-angle reflection is referred to as the forward-scattering component, and the light that undergoes large-angle reflection is referred to as the backward-scattering component. Therefore, without considering water absorption, the irradiance formula can also be written as

(2) $$E_{T}=E_{d}+E_{fs}+E_{bs}$$

where $E_{T}$ is the total irradiance emitted by the light source, $E_{d}$ represents the direct component, $E_{fs}$ represents the forward scattering component, and $E_{bs}$ represents the backward scattering component.

Scattering effects cause complex transformations in the propagation of light beams, resulting in changes in the spatial and temporal distribution of light energy [13]. The causes of scattering effects are impurity particles in the water, and there are many impurity particles present, with various sizes. Therefore, the impact of scattering effects on the quality of underwater images is significant, mainly manifested in three aspects. Firstly, the loss of light energy reduces the detectable distance of the targets. Secondly, backward-scattered light generates a large amount of noise on underwater images, affecting the signal-to-noise ratio of the images. Thirdly, forward-scattered light disperses light energy, resulting in image blur, the softening of contours, and reduced visual effects.

Since the attenuation of light transmission underwater and backward scattering are the primary factors limiting the operational distances of underwater electro-optical imaging systems, reducing backward scattering and enhancing the image contrast have become the focus of research in underwater electro-optical imaging technology.

### 2.2. Theory for SPAD Simulation

Underwater SPAD imaging utilizes the high sensitivity and time resolution capabilities of single-photon avalanche diodes (SPADs) to detect the arrival time of individual photons, achieving high-resolution imaging. This technology employs pulsed lasers as the light source, operating in the blue–green wavelength range (450–550 nm) to minimize light attenuation and scattering in water. By recording the arrival time of each photon and performing a statistical analysis, it can reconstruct two-dimensional or three-dimensional images of the target. This imaging technique works effectively in low-light environments and is suitable for marine exploration, biological research, and underwater archaeology, overcoming the light propagation limitations faced by traditional underwater imaging methods.

**Figure 2:**
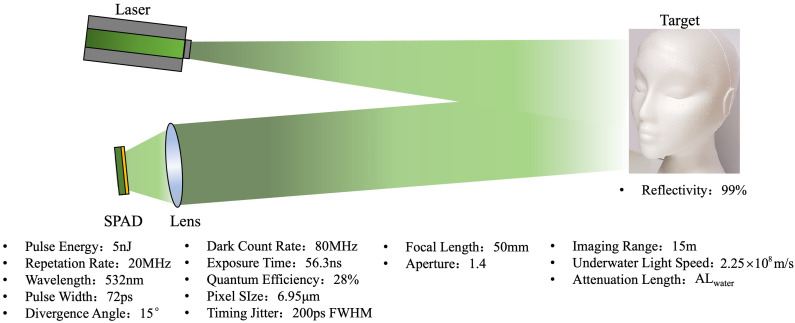
Schematic of underwater SPAD imaging system.

The number of photons from the target is described by the photon channel, which models the loss of signal photons as a series of continuous processes. Consider a laser pulse with wavelength λ, initial energy $E_{0}$, and divergence angle θ, passing through an underwater medium with attenuation length $AL_{water}$ and projected onto a target with range *R*, as shown in Figure 2. The energy density $\rho_{E}$ at the target is given by

(3) $$\rho_{E}=\frac{E_{0}e^{\frac{-R}{AL_{water}}}}{\pi R^{2}\tan^{2}\left(\theta \right)}$$

For an imaging sensor with effective dimensions of $W_{p}$ × $H_{p}$ pixels, located on the focal plane of a collection lens with focal length *f* and aperture number $f_{no}$, the energy $E_{1}$ that each pixel can receive is given by

(4) $$E_{1}=\rho_{E}\left(\frac{R^{2}W_{p}H_{p}}{f^{2}}\right)$$

Assuming that the target object is a Lambertian reflector, for a target object with reflectivity Γ, the energy $E_{2}$ reaching each pixel of the lens aperture after reflection is given by

(5) $$E_{2}=\frac{\Gamma E_{1}e^{\frac{-R}{AL_{water}}}}{2\pi R^{2}}$$

Then, based on the aperture of the lens and the quantum efficiency *q* of the SPAD detector, a portion of this energy $E_{2}$ is captured by each pixel, denoted as $E_{3}$:

(6) $$E_{3}=qE_{2}\pi {\left(\frac{f}{2f_{no}}\right)}^{2}$$

Combining Equations (3)–(6) and dividing the result by the unit photon energy hc/λ, where λ is the wavelength of the illuminating light, the number of photons captured by the detector per pulse, denoted as $P_{pp}$, is given by

(7) $$P_{pp}=\frac{\lambda E_{0}}{hc}\frac{q\Gamma e^{\frac{-2R}{AL_{water}}}}{8}\frac{W_{p}H_{p}}{f_{no}^{2}\pi R^{2}tan^{2}\left(\theta \right)}$$

in which *h* represents Planck’s constant, and *c* represents the speed of light. The value of 8 in the denominator treats surface reflectance Γ and backward scattering underwater $AL_{water}$ as the outcomes of two independent processes. The value in the denominator will change if different scattering mechanisms were considered. However, even if *P_pp_* > 1, due to the per-pulse single-photon constraint of SPAD, only a single photon would be measured. In fact, for many SPAD imaging applications, the number of photons detected per pulse per pixel is less than 1. Therefore, Equation (7) represents the probability of signal photons being measured by the SPAD detector. In other words, one can expect the SPAD to be triggered at least once on average every 1/*P_pp_* pulses.

Additionally, we assume that Equation (7) applies only for a single wavelength. Generally, Equation (7) can be extended to multiple wavelengths by integrating over all relevant *λ*. Equation (7) is a variant of the radar response equation, where Γ acts as the optical cross-section, and $\left(W_{p}\times H_{p}\right)/\left[f_{no}^{2}\pi R^{2}\tan^{2}\left(\theta \right)\right]$ defines the relationship between the effective aperture and the illuminated area. This ratio suggests that larger pixels in the SPAD array detector are preferable because, within the limit of a single-point detector, it can observe the entire field of view, and all collected energy will be received by one pixel.

### 2.3. Simulation of SPAD Depth Map

Inspired by the excellent work of Scholes S. et al. [14], we design our underwater SPAD simulation based on Fisher information. Fisher information is a crucial concept in statistics, used to measure the uncertainty about a parameter in probability distribution parameter estimation. The introduction of Fisher information aims to simulate the uncertainty in the photon arrival times. Based on the true value of the photon arrival time benchmark, by incorporating Fisher information, the simulated data can be made more realistic.

Assuming that the pulse response functions of the laser pulse and SPAD are approximately Gaussian in time, with a mean of μ and a standard deviation of $\sigma^{\prime }$, Equation (7) can be generalized to a likelihood function:

(8) $$L\left(t|\mu ,\sigma^{\prime }\right)=C_{dc}+C_{bksct}+\frac{P_{pp}}{\sigma^{\prime }\sqrt{2\pi }}\exp\left[-\frac{1}{2}{\left(\frac{t-\mu }{\sigma^{\prime }}\right)}^{2}\right]$$

where $C_{dc}$ is the dark count rate of the SPAD detector, and $C_{bksct}$ is the backward-scattering count rate caused by the underwater scattering environment. It is assumed that both rates are constant over time.

Then, by performing TCSPC probability integration over the time interval [0, T] for Equation (8), we can obtain the average number of photons measured by the detector for each pulse and pixel within this interval, which is α.

(9) $$\begin{array}{cc} \; & F\left(t|\mu ,\sigma^{\prime }\right)=\int_{0}^{T}\left\{\frac{\partial \ln\left[\frac{L(t|\mu ,\sigma^{\prime })}{\alpha }\right]}{\sigma \mu }{\}}^{2}\frac{L(t|\mu ,\sigma^{\prime })}{\alpha }dt\right.\\ \; & =\int_{0}^{T}\frac{P_{pp}^{2}{\left(t-\mu \right)}^{2}\exp\left[-{\left(\frac{t-\mu }{\sigma^{\prime }}\right)}^{2}\right]}{{\sigma^{\prime }}^{6}2\pi \alpha \left\{C_{dc}+C_{bksct}+\frac{P_{pp}}{\sigma^{\prime }\sqrt{2\pi }}\exp\left[-\frac{1}{2}{\left(\frac{t-\mu }{\sigma^{\prime }}\right)}^{2}\right]\right\}}dt \end{array}$$

Equation (9) is a problem without a closed-form analytical solution. However, we can create a photon arrival time histogram by accumulating frames. Furthermore, when estimating the peak position of a histogram, the Cramer–Rao bound defines the lower bound on the standard deviation $\sigma_{\mu }^{*}$, which characterizes the minimum possible standard deviation associated with estimating the depth of a single point.

(10) $$\sigma_{\mu }^{*}=\frac{1}{\sqrt{N\left[1-{\left(1-\alpha \right)}^{\eta \nu }\right]F\left(t|\mu ,\sigma^{\prime }\right)}}$$

In this equation, *N* represents the number of frames, η represents exposure time, ν represents laser repetition rate, and 1−(1−α) represents the probability that at least one photon can be measured in this frame. *N*[1−(1−α)ην] represents the number of successful events recorded in the histogram. Equation (10) represents the minimum possible standard deviation in estimating the depth of a single point. Through Equations (7)–(10), the influence of a single optical parameter on the variance of depth estimation can be separated. Specifically, changing parameters such as the scattering rate of the medium will not affect other unrelated parameters, leading to bias in depth estimation. When $P_{pp}=0$, through Equation (9), it can be seen that the Fisher information is zero, which is consistent with the concept that the depth of the target object cannot be inferred from the dark count of SPAD and the backward-scattered photons from the water. We can directly manipulate the Fisher information by adjusting the given signal-to-back-scattering-noise ratio (SbNR):

(11) $$\mathrm{SbNR}=\left[E_{0}\exp\left(-R/AL_{water}\right)\right]/\left[W_{bksct}\pi R^{2}\tan^{2}\left(\theta \right)\right]$$

In this context, $W_{bksct}$ represents the radiant intensity of backward scattering from the water body. The Fisher information is strongly influenced by the pulse width $\sigma^{\prime }$. This strong control, along with the dependency of $E_{0}$ in the SbNR, means that, under the same average power, a laser illuminator with higher peak power will exhibit a superior imaging performance. Lastly, the system’s performance will be affected by the change in the laser repetition rate ν in a manner similar to the change in parameters. This is consistent with the fact that the imaging performance of a laser radar system depends on the parameter.

Equation (10) describes the lower bound of the standard deviation in the estimation of the peak position of the photon arrival time in the histogram. Therefore, by obtaining the true depth value of a scene, we can obtain a realistic SPAD-simulated image through estimation methods.

Assuming that the true depth value of a target object’s depth map is μ, then the true depth value at the (m,q) pixel of this depth map is $\mu_{\left(m,q\right)}$, so that it can be $\mu_{\left(m,q\right)}\to \mu_{\left(m,q\right)}+\Delta \mu$, where Δμ is a sample value from a normal distribution with a mean of 0 and a minimum standard deviation of $\sigma_{\mu }^{*}$.

### 2.4. Simulation of Backward Scattering

The Monte Carlo simulation is a method of obtaining results by conducting a large number of experiments in a simulated environment. As long as the sample size is sufficiently large, a relatively accurate result can be obtained. This is particularly advantageous in complex environments, as it eliminates the need to individually analyze each influencing factor and its weighting. Instead, one only needs to design a simulated environment that reflects reality and then conduct multiple experiments [15].

The principle of the Monte Carlo method is simple, but its practical application is challenging due to the high demands for experimentation. With advancements in technology, the powerful capabilities of computers have revitalized the Monte Carlo method. Nowadays, the Monte Carlo method is applied in various fields with complex background environments, and ocean optical detection is one of them. The composition of seawater is complex, and light may interact with various particles during transmission, undergoing multiple scattering events. These particles vary in size, shape, and light absorption characteristics. Even for spherical particles, it is difficult to obtain solutions for multiple scattering using the Mie scattering theory. However, the Monte Carlo method can avoid these complex calculations. By conducting numerous simulation experiments on computers, ideal results can be obtained.

A beam of light consists of a large number of photons. By using photons as the basic units of experimentation and simulating the transmission of a large number of photons in water, one can analyze the diffusion of the light beam, the distribution and attenuation of its energy, and the effective propagation distance of the light beam. Suppose that the position of a photon at a certain moment is $\left(x_{n},y_{n},z_{n}\right)$, and, after undergoing a scattering event, the coordinates become $\left(x_{n+1},y_{n+1},zn+1\right)$. The relationship between them satisfies the following:

(12) $$\left\{\begin{array}{c} x_{n+1}=x_{n}+lu_{x}\\ y_{n+1}=y_{n}+lu_{y}\\ z_{n+1}=z_{n}+lu_{z} \end{array}\right.$$

In the equation, *l* represents the mean free path of the photon’s motion. According to the Lambert–Beer law of light absorption in water, *l* can be calculated using the following equation:

(13) $$l=-\frac{1}{c}\ln\zeta_{1}$$

*c* is the attenuation coefficient of light in water, which is the sum of the absorption coefficient and the scattering coefficient; $\zeta_{1}$ is a uniformly distributed random number in the range (0,1). $u=(u_{x},u_{y},u_{z})$ is the unit vector representing the direction of photon motion.

Based on the description of the Hengey–Greenstein phase function, we can determine the angle between the trajectories of a photon and underwater particles before and after collision. However, this only defines a cone, and an azimuth angle is needed to determine the specific direction. Based on the assumption of collisions with spherical particles, this azimuth angle is random and can be defined as follows:

(14) $$\phi =2\pi \zeta_{2}$$

$\zeta_{2}$ is a uniformly distributed random number in the range (0,1). With the direction and displacement, the state of the photon after each collision can be determined. The new direction $u_{n+1}$ of the photon after collision is

(15) $$\left\{\begin{array}{c} u_{x,n+1}=\frac{sin\theta_{n}}{\sqrt{1-u_{z,n}^{2}}}\left(u_{x,n}u_{z,n}cos\phi_{n}-u_{y,n}sin\phi_{n}\right)+u_{x,n}cos\theta_{n}\\ u_{y,n+1}=\frac{sin\theta_{n}}{\sqrt{1-u_{z,n}^{2}}}\left(u_{y,n}u_{z,n}cos\phi_{n}+u_{x,n}sin\phi_{n}\right)+u_{y,n}cos\theta_{n}\\ u_{z,n+1}=-sin\theta_{n}cos\phi_{n}\sqrt{1-u_{z,n}^{2}}+u_{z,n}cos\theta_{n} \end{array}\right.$$

When the direction of photon motion is very close to the *z*-axis, the new direction $u_{n+1}$ is

(16) $$\left\{\begin{array}{c} u_{x,n+1}=sin\theta_{n}cos\phi_{n}\\ u_{y,n+1}=sin\theta_{n}sin\phi_{n}\\ u_{z,n+1}=\frac{u_{z,n}}{|u_{z,n}|}cos\theta_{n} \end{array}\right.$$

**Figure 3:**
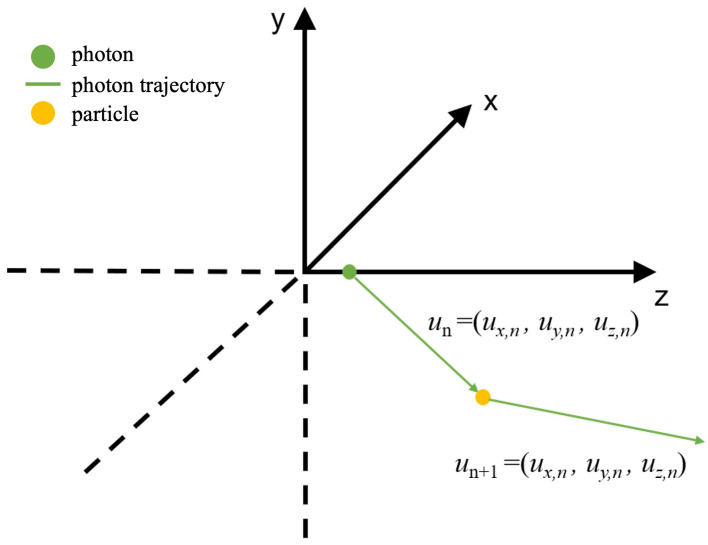
Schematic diagram of single-photon scattering.

We can set $u_{z,n}\geq$ 0.9998 to satisfy the condition of being close to the z-axis. The directional relationships described above are illustrated in Figure 3, where the green circle represents a photon at a beginning point, the green arrow represents the trajectory of a photon moving in underwater environment, and the yellow particle represents the particle in water.

Light propagation in water undergoes losses, manifested microscopically as the continuous attenuation of the photon energy until it becomes undetectable. Additionally, deviations in the photon motion direction due to scattering can lead to photons not being received by detectors, which can also be considered to be photon disappearance. A summary is given below.

Loss of photon energy propagation: The underwater environment is filled with water molecules; even if a photon does not collide but continues to propagate in a straight line, it will lose energy due to the interaction with water molecules. This situation is universal for all photons in optical detection, so it can be directly described by the macroscopic light energy loss, namely, the absorption coefficient, without the need for precision regarding each photon. The maximum propagation distance of the photons can be set based on this.

Loss of photon energy due to collision: Previously, various components and their content in seawater were analyzed. For photons, a single collision can significantly alter their energy. The diversity of the components in water means that the energy loss from each collision of a photon is different. For general particles causing light scattering, the scattering rate of a seawater medium can approximate the energy loss.

Deviation in photon motion: After collisions, the direction of photon motion may significantly deviate from the z-axis, ultimately causing the photon to become undetectable. In the context of detection, such photons are considered extinct. This condition can be implemented by programming the system to exclude photons with significantly large absolute values of the x and y coordinates.

Multiple collisions of photons: Photons that undergo multiple collisions may still retain a considerable amount of energy and be received by detectors. Considering this as a very low-probability event, and the significantly increasing computational complexity created by multiple collisions for the Monte Carlo method, which requires a large number of simulated photons, these photons need to be excluded. Thus, by setting a limit on the number of collisions, photons reaching this limit are considered extinct.

Based on the above assumptions, a simulation can be conducted. Single-scattering rate calculation is relatively complex, requiring the consideration of the particle type, size, and depth distribution. Models describing the particle size distribution include the hyperbolic, segmented hyperbolic, and two-component model (TCM), among others. The TCM fits well with seawater. In this model, the cumulative probability of the particle size follows a logarithmic curve, rapidly increasing from 0 to 20 μm and then remaining nearly constant. Within the range of 0–20 μm, the relationship between the particle size and cumulative probability is approximately linear, indicating an equal number of particles of various sizes, and, thus, equal probabilities of photons colliding with different-sized particles. Consequently, to simplify the above, the single-scattering rate in the program is set as uniformly distributed values between 0 and 0.97. Additionally, the attenuation coefficient is set to 0.035, and the asymmetry factor *g* of the H-G phase function is set to 0.924. Setting the initial energy weight of photons to 1, photons with energy weights below 0.0002 are considered extinct under the sole constraint of energy.

## 3. Method for Simulation of Underwater SPAD Dataset

### 3.1. Underwater Virtual Environment Setup

Synthetic data are often used to train machine learning models, helping them to understand and process data in different situations. Virtual engines can generate large amounts of rich synthetic data for model training, without the need to collect and annotate data from the real world, thus reducing the costs and risks. Mainstream virtual engine software includes Unity, Unreal Engine (UE), Blender, and Issac Sim.

**Figure 4:**
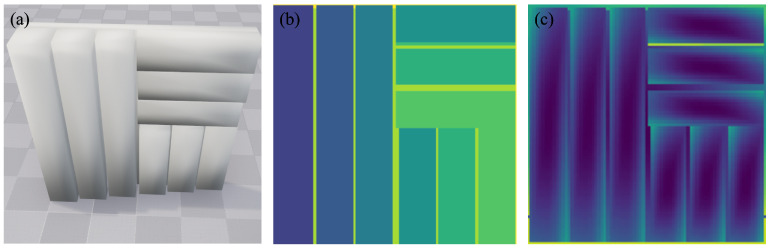
(**a**) A 3D target in UE. (**b**) Depth map of the target. (**c**) Surface normal of the target.

In our work, we chose UE5 as the dependent software to build the underwater virtual environment. We designed a three-dimensional target with dimensions of 400 × 400, as shown in Figure 4, with 3 groups of rectangular cuboids distributed on it. The left group contains 3 rectangular cuboids whose heights range from 40 cm to 60 cm. The upper right group and lower right group both contain 3 rectangular cuboids whose heights differ from 10 cm to 30 cm.

As for the SPAD camera, we created a sensor board inside a cuboid in UE. According to the functionalities of UE, it is possible to export the depth map, normal map, and base color of target objects.

**Figure 5:**
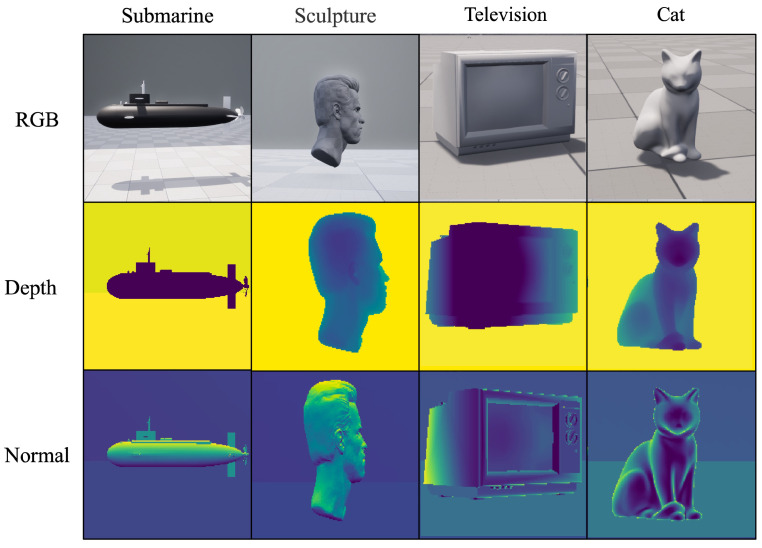
Samples of open-source assets in UE.

Besides the three-dimensional target that we designed, we also chose some open-source assets in UE [16] as targets. Some of the assets are shown in Figure 5.

### 3.2. System Parameter Settings

The principle of the underwater SPAD imaging system is to utilize single-photon avalanche diode detectors to detect photons reflected from the target object and then convert these detected photons into images using the imaging system. Due to the extremely high time resolution of SPADs, this imaging system is often used for time-resolved imaging, such as measuring the time of photon arrival to obtain distance information about the target object.

Considering the influence of underwater environments on light transmission, water molecules and dissolved substances in the environment have different absorption characteristics for light of different wavelengths. Comparatively, red and yellow light have higher energy and can penetrate deeper into seawater, but they are absorbed to a greater extent during the penetration process. In contrast, blue light has lower energy; although it displays strong scattering on the water surface, it can penetrate relatively further in deep water because it experiences less absorption. Therefore, a laser with a wavelength of 532 nm is chosen as the light source for the underwater SPAD imaging system design.

Regarding the parameters of the optical system, we selected a state-of-the-art laser, the PicoQuant VisUV, and SPAD camera, the Photon Force PF32, as references. The relevant parameters are as shown in Table 1 and Table 2. The parameters for the optical system are shown in Table 3.

### 3.3. Procedure of Underwater SPAD Simulation

**Figure 6:**
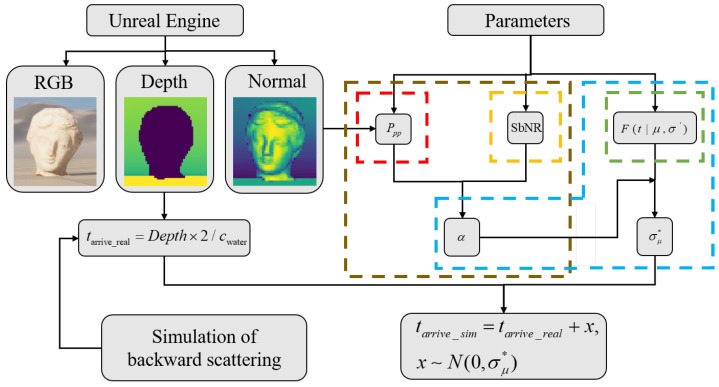
Flowchart of underwater SPAD simulation.

In summary, the procedure for the design of the underwater SPAD simulation is illustrated in Figure 6, with the specific steps outlined as follows.

(a) Using the virtual environment software Unreal Engine, construct a three-dimensional target object with a plastic material. Place a camera in the virtual environment at a distance of 15 m and 50 m, respectively, from the target and export the target’s RGB image, depth map, surface normal map, etc., from the software. The exported RGB image represents the intensity information ground truth of the target, the depth map represents the depth information ground truth of the target, and the surface normal map represents the material, reflectance, and other ground truths of the target.
(b) Multiply the values in the depth image by 2 and divide them by the underwater speed of light to obtain the theoretical ground truth of the time at which photons arrive at each pixel in the underwater environment. Since each pixel contains a TCSPC histogram channel, the ground truth of the photon arrival time is considered to be the position of the histogram peak.
(c) Substitute all known parameters into Equation (7) to obtain the number of photons detected per pulse. Calculate the proportion of back-scattered photons detected after each pulse using Equation (11).
(d) Use Equations (8) and (9) to calculate the Fisher information and the average number of photons measured per pixel within [0, T]. Calculate the lower bound of the standard deviation of the estimated peak position of the histogram arrival time using Equation (10), i.e., the Cramer–Rao bound.
(e) Add a random number following a normal distribution with a mean of 0 and a standard deviation of $\sigma \mu^{*}$ to each pixel’s ground truth of the photon arrival time. This yields the result of the underwater SPAD simulation.

The calculation results within the red dashed box in Figure 6 represent the results of Equation (7), those within the yellow dashed box represent the results of Equation (11), those within the green dashed box represent the results of Equation (9), those within the brown dashed box represent the results of Equation (8), and those within the blue dashed box represent the results of Equation (10). Ultimately, by combining the aforementioned theoretical results, the simulation results of underwater SPAD imaging are obtained. Through this simulation process, a large number of underwater SPAD imaging simulation images under different water quality and distance conditions can be obtained, providing training data for subsequent deep learning-based underwater SPAD three-dimensional image reconstruction algorithms.

**Figure 7:**
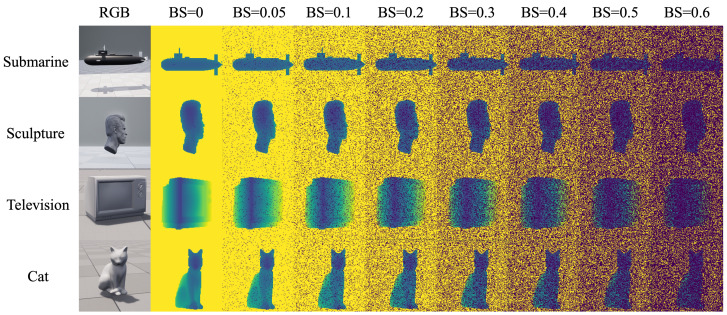
Samples of results of underwater SPAD simulation.

Figure 7 shows some results of the underwater SPAD simulation. BS represents the degree of backward scattering. As BS increases, more pixels on the simulated SPAD are triggered by the backward scattering of underwater particles, which is in line with the reality.

## 4. Practice Underwater SPAD Simulation Dataset

The underwater background light prior model is a model established based on the light propagation characteristics and background noise factors in the underwater environment. It is used to describe the statistical properties and distribution patterns of underwater background light. This model is typically based on factors such as the water quality, depth, and water type, considering optical phenomena such as scattering, absorption, and reflection in water, as well as the influence of suspended particles and organisms in the water on the light field. The establishment of the underwater background light prior model helps in understanding the distribution of the light fields in the underwater environment, providing important references for underwater image processing and computer vision tasks.

### 4.1. Denoising Network

In order to demonstrate the effectiveness of our data, we utilized the Underwater SPAD Denoise Network (USDN), an algorithm based on DehazeNet [17], to train a denoising model for underwater SPAD images. Subsequently, we validated the model using real underwater SPAD data.

In the field of image dehazing, DehazeNet is a classical model for the enhancement of images corrupted by atmospheric scattering. DehazeNet is an end-to-end deep learning approach specifically designed for real-time single-image dehazing, aiming to recover unknown clear images from those affected by haze. The reason why DehazeNet is chosen is that DehazeNet is a simple yet effective CNN model which considers the physics prior to underwater light transmission. Therefore, it is different from other underwater image recovering or dehazing models that only consider color recovery. This method is crucial in improving the image quality under haze effects, which is essential for various image analysis tasks, including object detection and scene segmentation. Preprocessing images to enhance their quality can enhance the performance in these tasks.

**Figure 8:**
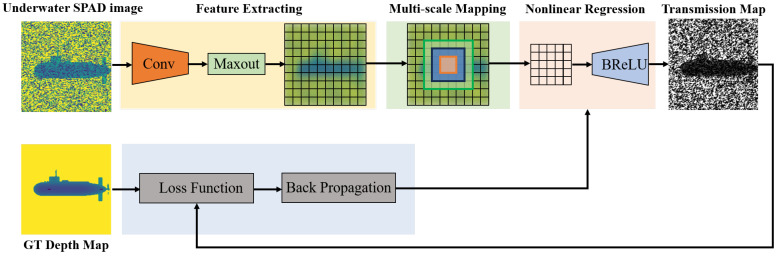
Structure of USDN based on DehazeNet.

Since the imaging model for underwater imaging is fundamentally similar to that of haze imaging, with differences mainly in the concentration, absorption coefficient, and scattering coefficient of the backward-scattering medium, we considered building upon DehazeNet to design the USDN. The network structure of the USDN is illustrated in Figure 8.

### 4.2. Metrics

We used underwater SPAD simulation data obtained at different concentrations of backward scattering as input to the network. We extracted the features of the underwater SPAD images through a feature extraction network; extracted features related to underwater interference factors through multiscale mapping; and obtained the predicted transmittance and scattering rate maps through nonlinear regression. During training, we utilized the mean squared error (MSE) as the loss function and the peak signal-to-noise ratio (PSNR) as the evaluation metric on the validation set. Moreover, to validate the effectiveness of the denoising, we employed the PSNR, Structural Similarity Index Metric (SSIM), and image entropy as evaluation metrics.

The peak signal-to-noise ratio (PSNR) [18] values indicate the similarity between the noisy image and the clean image. A higher PSNR value suggests that the noisy image is closer to the original clean image, implying lower noise levels and clearer images. The formula for the calculation of the PSNR is

(17) $$\mathrm{PSNR}=10\cdot \log_{10}\left(\frac{{\mathrm{MAX}}^{2}}{\mathrm{MSE}}\right)$$

where MAX represents the maximum possible value in the signal, which is 255 for an 8-bit digital signal. The denominator MSE represents the mean squared error, which is the mean of the squared differences between the original and corrupted signals. The formula for the calculation of the MSE is

(18) $$\mathrm{MSE}=\frac{1}{N}\sum_{i=1}^{N}{\left(x_{i}-y_{i}\right)}^{2}$$

where $x_{i}$ is the *i*th sample value of the original signal, $y_{i}$ is the *i*th sample value of the corrupted signal, and *N* is the number of samples.

The SSIM is a metric used to evaluate the quality of images, taking into account aspects such as the overall grayscale, contrast, and structural similarity of the image [19].

(19) $$\mathrm{SSIM}\left(x,y\right)=\frac{\left(2\mu_{x}\mu_{y}+c_{1}\right)\left(2\sigma_{xy}+c_{2}\right)}{\left(\mu_{x}^{2}+\mu_{y}^{2}+c_{1}\right)\left(\sigma_{x}^{2}+\sigma_{y}^{2}+c_{2}\right)}$$

where *x* and *y* represent the two images being compared, $\mu_{x}$ and $\mu_{y}$ represent the mean grayscale values at local positions in the two images, $\sigma_{x}$ and $\sigma_{y}$ represent the standard deviations of the grayscale values at local positions in the two images, $\sigma_{xy}$ represents the covariance of the grayscale values at local positions in the two images, and $c_{1}$ and $c_{2}$ are constants. The SSIM can be considered as a probability value, ranging from −1 to 1, where values closer to 1 indicate greater similarity between the two images.

Image entropy is a metric used to measure the amount of information in an image, reflecting the uncertainty or randomness of the pixel values in the image [20]. Higher entropy values indicate a more random distribution of pixel values and greater information content, while lower entropy values indicate a more concentrated distribution of pixel values and less information.

(20) $$EN\left(x\right)=-\sum_{i=1}^{L}p\left(x_{i}\right)\log p\left(x_{i}\right)$$

where EN(x) represents the image entropy, *L* is the number of grayscale levels in the image, $x_{i}$ represents the grayscale value of each pixel in the image, and *p*(*x_i_*) is a probability value representing the probability of occurrence of *x_i_* in the image.

### 4.3. Results and Analysis

**Figure 9:**
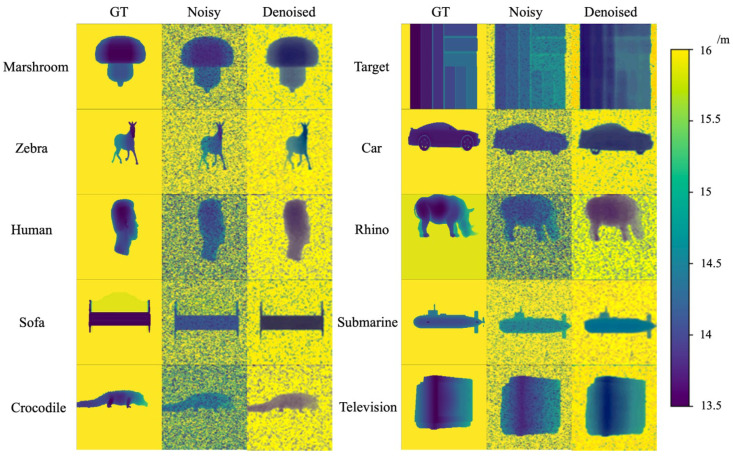
Samples of the results of the USDN inferring on simulated data.

We train the USDN with a simulated underwater SPAD dataset. The dataset contains 20 different target objects. By simulating the underwater SPAD depth imaging of 20 target objects, we obtained 1000 simulated depth maps for each object at various attenuation lengths. To avoid overfitting due to limitations in features such as target object contours and shapes, and to expand the training dataset, we augmented the 20,000 frames of the simulated underwater SPAD data via random cutting and rotation. Finally, the dataset for training contained 400,000 pairs of clean (ground truth) and noisy images. Some of the results of the USDN are shown in Figure 9.

From the results, it can be seen that the noise in the background part of the denoised image is smoothed, and the noise on the target objects is completely eliminated, which proves the effectiveness of the USDN model in denoising. Furthermore, in the extracted samples, the PSNR after denoising is higher than that before denoising, with an average increase of 5.59 dB. To further analyze the denoising performance of the USDN model, the evaluation metrics described in Section 4.2 are used to measure its denoising effect.

**Figure 10:**
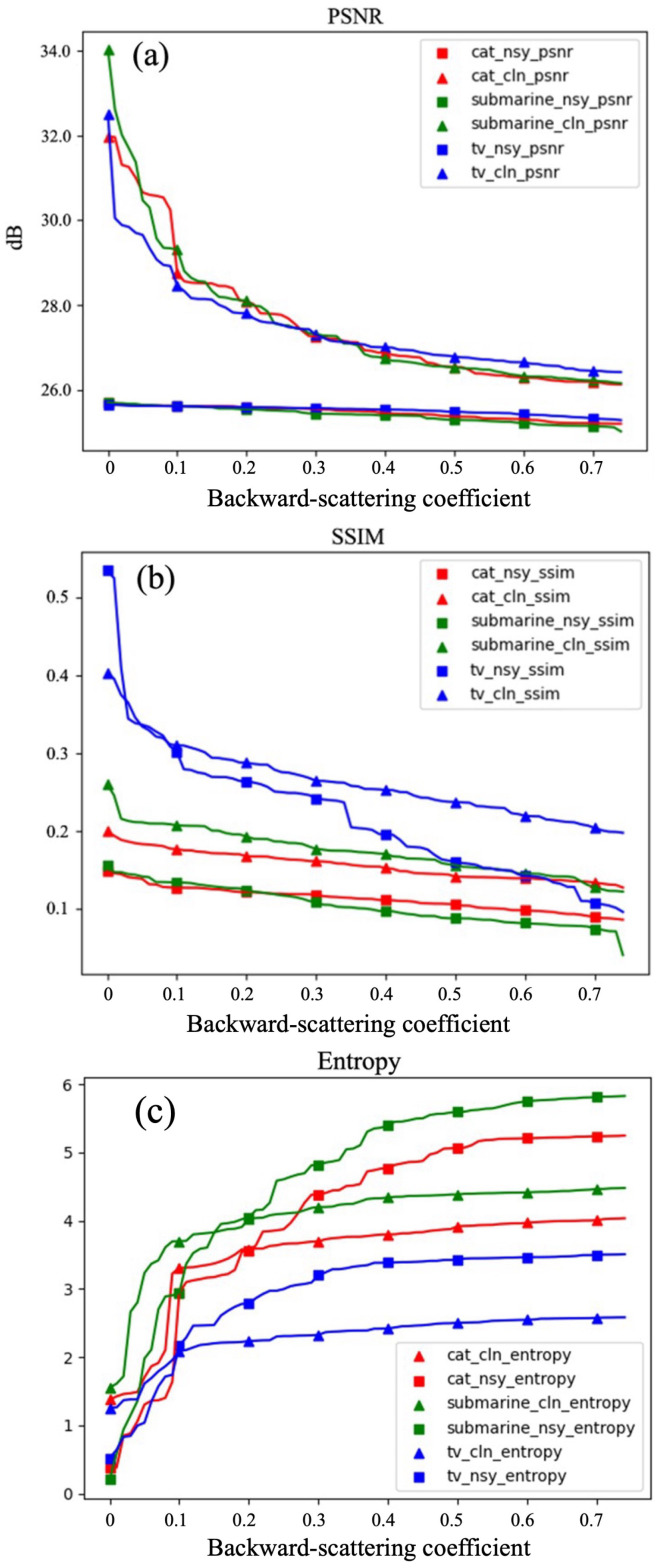
(**a**) PSNR results of USDN inferencing on simulated data. (**b**) SSIM results of USDN inferencing on simulated data. (**c**) Entropy results of USDN inferencing on simulated data.

We select three target objects, which are cat, submarine, and television, as demonstrations. The results are shown in Figure 10. In Figure 10, the horizontal axis represents the backward-scattering coefficient, and the vertical axis represents the value of metrics. In the legend, ‘nsy’ denotes the image before denoising, and ‘cln’ denotes the image after denoising.

The PSNR results are shown in Figure 10a. It can be clearly observed that the PSNR of the denoised images is higher compared to the original noisy images. An increase in the PSNR typically indicates an improvement in image quality. A higher PSNR implies lower distortion in the image, making it closer to the original signal. On average, the PSNR is increased by 5.59 dB after denoising compared to that before denoising.

The SSIM results are shown in Figure 10b. It is found that the structural similarity of the denoised images is improved compared to the original noisy images. This indicates that the denoised images exhibit a high degree of similarity to the ground truth (GT) in terms of structure, with minimal differences. Therefore, it can be inferred that there is no significant distortion between the images, or the distortion is very slight. On average, the SSIM is increased by 9.03% after denoising compared to that before denoising.

The entropy results are shown in Figure 10c. According to the explanation in Section 4.2, if the entropy of the denoised image is close to the entropy of the original image, it indicates that the denoising algorithm effectively preserves the structural information of the image while removing noise. As shown in Figure 10c, when the noise level is low in the first 10 frames, the distance between the entropy of the original image and the GT is closer compared to the distance between the entropy of the denoised image and the GT, and the situation reverses after 10 frames. Therefore, it can be inferred from the perspective of image entropy that the USDN model is more suitable for denoising tasks with high noise levels. On average, the image entropy is decreased by 0.84 after denoising compared to that before denoising.

#### 4.3.1. Visual Analysis

**Figure 11:**
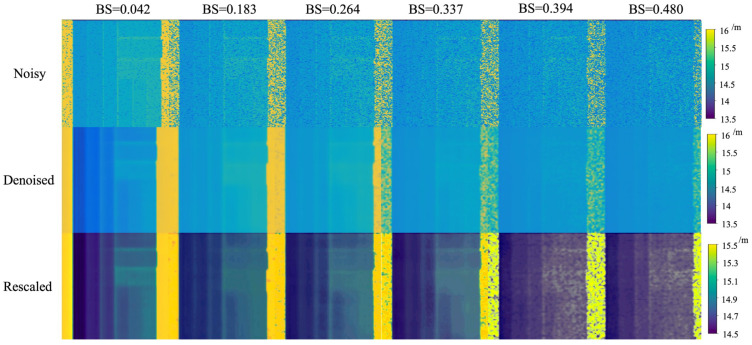
Denoising results at different backward-scattering levels.

The inference results of the USDN in underwater SPAD images at different attenuation lengths are shown in Figure 11. The figure lists the underwater SPAD simulation data obtained with different backward-scattering coefficients (BS = 0.042, BS = 0.183, BS = 0.264, BS = 0.337, BS = 0.394, BS = 0.480), along with their corresponding USDN inference results. Comparing the noisy and denoised images, it can be clearly seen that the noise in the images has been reduced, and the images have become smoother. However, as the backward-scattering coefficient increases, some details of the targets gradually disappear. To avoid the problem of detail loss caused by the excessively large range of pseudocolorization, the inference results were revisualized, namely, rescaled, to control the pseudocolorization within a smaller range, thereby obtaining richer detail information. From the rescaled images, it can be seen that the inference results in the first four columns retain the detail information of the original image, and the entire staircase on the target can be distinguished. In the last two columns, due to the large backward-scattering coefficient, the model removes some detail information during the denoising process, resulting in a poorer effect, but some staircases can still be distinguished.

**Figure 12:**
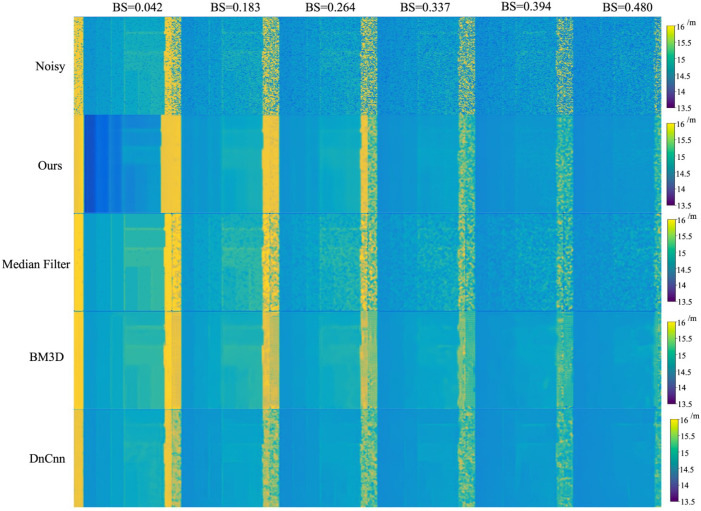
Comparison of several common image denoising algorithms.

**Figure 13:**
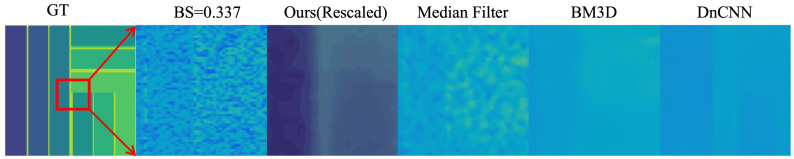
Comparison of depth map details.

To further demonstrate the performance of the USDN, we compare several common image denoising algorithms, including Median Filtering [21], BM3D [22], and DnCNN [23]. The comparison results are shown in Figure 12, where we use the PSNR, SSIM, and entropy as evaluation metrics, and the results are presented in Table 4. The comparison of the depth map details is illustrated in Figure 13.

In Table 4, it can be observed that the USDN performs the best across all three metrics, followed by DnCNN and BM3D, while the median filtering algorithm performs the worst. DnCNN is a supervised image denoising algorithm based on convolutional neural networks, and BM3D is a traditional denoising algorithm designed for additive Gaussian noise. As shown in Figure 12 and Figure 13, the USDN maintains good performance across a range of noise levels, from low to high; the median filtering method does not completely remove the noise; BM3D performs well at low noise levels but causes the blurring of image details at high noise levels; and DnCNN confuses deep details during denoising, leading to a decrease in the denoising accuracy.

#### 4.3.2. Inference on Real Underwater SPAD Data

**Figure 14:**
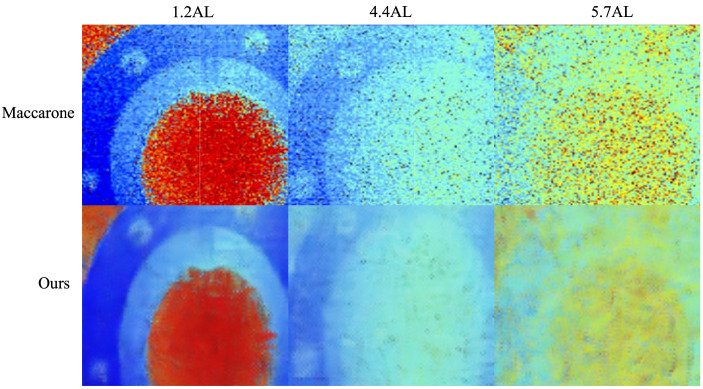
USDN inference on real underwater SPAD data.

To validate the effectiveness of the USDN, it is necessary to perform inference on real underwater SPAD data and verify the model’s performance. Since Maccarone et al. have conducted a series of experiments and image processing work in the field of underwater SPAD imaging [5], we chose to validate our method using the experimental data from Maccarone’s team. The results are shown in Figure 14.

From the analysis of Figure 14, it can be seen that the USDN achieved good results at underwater attenuation lengths of 1.2 and 4.4, with most of the back-scattering noise removed from the images. However, at 5.7AL, although the USDN effectively removed most of the noise, it only restored the overall outline of the target object, without providing more details.

To further quantitatively analyze the performance of the USDN on real data, we use evaluation metrics for analysis. Since Maccarone did not provide ground truth depth maps, the PSNR and SSIM cannot be used to evaluate the model performance. However, since the entropy does not require reference images, we use the entropy for evaluation. We calculated the metrics of the USDN’s inference results on real data relative to Maccarone’s images. The results are shown in Table 5.

In Table 5, it can be observed that, compared to Maccarone’s denoising algorithm, the USDN shows a decrease in image entropy at all three attenuation lengths, indicating an improvement in image quality and demonstrating the effectiveness of the USDN’s inference on real data.

## 5. Discussion

The main focus of this study is the degradation of images in underwater environments due to the problem of backward-scattering interference in single-photon imaging. This study employs a deep learning approach to explore a new solution for the denoising of underwater single-photon avalanche diode (SPAD) images and proposes a Monte Carlo simulation-based method of obtaining underwater SPAD depth images and Time-Correlated Single-Photon Counting (TCSPC) histogram data. This method provides training data for underwater SPAD image denoising algorithms. The main contributions and innovations of this study are as follows.

1. We propose a simple and efficient simulation method for underwater SPAD depth images and TCSPC histograms. Addressing the difficulties in underwater SPAD imaging experiments and the high cost of SPAD devices, this study utilizes the Monte Carlo simulation to obtain underwater SPAD data. By combining the underwater light transmission model based on Fisher information estimation with the Monte Carlo simulation of underwater backward scattering, virtual environment software is used to obtain information about target objects, thereby generating a large amount of generalized underwater SPAD data. In the experiments, a dataset containing 20 target objects and 20,000 frames of depth images under different water scattering concentrations, as well as several frames of histogram data, is successfully obtained.
2. We propose the USDN, a deep learning-based denoising network for underwater SPAD. In underwater environments, due to the influence of backward-scattered light from impurity particles in water, SPAD devices are susceptible to receiving backward-scattered photons, triggering responses. In this scenario, the SPAD depth images and histograms obtained through scattering media carry a large number of noise signals, leading to decreased image contrast, making it difficult for traditional image denoising algorithms to effectively remove these interferences. Deep learning is an effective method to address this issue. This study simplifies the problem of removing backward-scattering interference in underwater SPAD images to a supervised deep learning model. By continuously allowing the neural network to learn the characteristics of backward-scattering noise based on prior knowledge of the underwater background light, the model can effectively remove the backward-scattering interference to obtain clear and restored images. The experimental results show that the USDN improves the peak signal-to-noise ratio (PSNR), Structural Similarity Index (SSIM), and entropy by 5.59 dB, 9.03%, and 0.84, respectively. The minimum depth resolution of the denoised underwater SPAD images can reach 10 cm.

## Figures and Tables

**Table 1:** Main parameters of PicoQuant VisUV.

Parameter	Value	Parameter	Value
Wavelength	532 nm	Average power	300 mW
Repetition rate	20 MHz	Pulse energy	5 nJ
Pulse width	<85 ps	Beam diameter	2.1±0.2 mm

**Table 2:** Main parameters of PhotonForce PF32.

Parameter	Value	Parameter	Value
Sensor size	32 × 32	Fill factor	20%
Photon efficiency	28%@500 nm	Bin width	55 ps
Max FPS	500 kHz	Jitter	<200 ps FWHM
Dark count rate	<100 Hz		

**Table 3:** Parameters for optical system.

Parameter	Value	Parameter	Value
Focal length	150 mm	F number	1.8
Target range	15 m	Target reflectance	78%
Backward scattering coefficient	0→0.97	Attenuation coefficient	0.035

**Table 4:** Comparison of PSNR, SSIM, and entropy. (↑ means metrics value increasing, and ↓ means metrics value decreasing).

	Ours	Median Filter	BM3D	DnCNN
PSNR	**5.59 ↑**	4.2 ↑	5.18 ↑	4.73 ↑
SSIM	**9.03% ↑**	3.41% ↑	6.84% ↑	8.72% ↑
Entropy	**0.84 ↓**	0.51 ↓	0.59 ↓	0.73 ↓

**Table 5:** Entropy of USDN inference on real underwater SPAD data.

	1.2AL	4.4AL	5.7AL
Entropy	0.31 ↓	0.57 ↓	1.22 ↓

## Data Availability

Dataset available on request from the authors.
